# Association Between Gensini Score and Left Ventricular Thrombus in Patients With Acute ST-Segment Elevation Myocardial Infarction

**DOI:** 10.31083/RCM48635

**Published:** 2026-06-25

**Authors:** Yang You, Hongliang Zhao, Haijiao Jin, Xiaoyan Han, Zelong Cao, Jun Liu, Guoqing Qi, Gang Liu, Mingqi Zheng

**Affiliations:** ^1^Department of Cardiology, The First Hospital of Hebei Medical University, 050000 Shijiazhuang, Hebei, China; ^2^Hebei Key Laboratory of Heart and Metabolism, 050031 Shijiazhuang, Hebei, China

**Keywords:** Gensini score, left ventricular thrombus, acute ST-elevation myocardial infarction, risk factors, coronary angiography

## Abstract

**Background:**

Left ventricular thrombus (LVT) is a serious complication of acute ST-segment elevation myocardial infarction (STEMI). Early risk stratification remains challenging. The Gensini score, quantifying coronary artery disease burden from angiography, may offer early prognostic value.

**Methods:**

This retrospective cohort study included 144 patients with a first STEMI treated by primary percutaneous coronary intervention. Patients were stratified into LVT (n = 36) and non-LVT (n = 108) groups based on transthoracic echocardiography. Clinical, laboratory, echocardiographic, and angiographic data were analyzed. The Gensini score was calculated, and multivariable logistic regression and receiver operating characteristic (ROC) curve analyses were performed.

**Results:**

Patients with LVT had a significantly higher median Gensini score (101.0 vs. 46.0, *p* < 0.001), alongside elevated B-type natriuretic peptide (BNP), reduced left ventricular ejection fraction (LVEF), and a higher incidence of left ventricular aneurysm. In multivariable analysis, a higher Gensini score (adjusted odds ratio [OR]: 1.47 per 10-unit increase, 95% CI: 1.21–2.62, *p *= 0.015), elevated BNP (OR: 1.32 per 100 pg/mL increase, 95% CI: 1.19–2.05, *p* = 0.040), reduced LVEF (OR: 0.52 per 5% increase, 95% CI: 0.30–0.84, *p* = 0.010), and presence of left ventricular aneurysm (OR: 2.46, 95% CI: 1.28–4.67, *p* = 0.006) were independently associated with LVT formation. ROC analysis identified an optimal Gensini score cut-off of 72.5 (AUC 0.785) for identifying LVT risk.

**Conclusion:**

The angiographic Gensini score is independently associated with LVT formation post-STEMI. Readily available after primary PCI, it enhances early risk stratification, potentially guiding intensified surveillance and preventive strategies for high-risk patients.

## 1. Introduction

Despite significant advancements in reperfusion strategies and adjunctive antithrombotic therapies, left ventricular thrombus (LVT) remains a formidable complication following acute ST-segment elevation myocardial infarction (STEMI) [1,2]. Recent scientific statements and guidelines highlight the complexity of managing patients at risk for and with LVT, emphasizing the need for improved risk stratification [3,4]. Modern primary percutaneous coronary intervention (PCI) has markedly reduced the incidence of LVT, yet contemporary meta-analyses report a prevalence of approximately 6.3% in the overall STEMI population, escalating to 12.2% in patients with anterior wall myocardial infarction (MI) and as high as 19.2% in those with a left ventricular ejection fraction (LVEF) below 50% [5,6]. The clinical sequelae of LVT are severe, primarily driven by systemic thromboembolism, which confers a substantial risk of stroke and increased mortality [7,8,9].

The pathogenesis of LVT is classically attributed to Virchow’s triad: endothelial injury, blood stasis, and a hypercoagulable state [10,11]. In the context of STEMI, extensive transmural necrosis leads to endocardial damage and inflammation, while severe regional wall motion abnormalities promote intracavitary blood stasis [12]. Concurrently, the systemic inflammatory response to myocardial injury fosters a prothrombotic milieu [13]. Consequently, established risk factors for LVT predominantly revolve around the consequences of a large myocardial infarct, including anterior infarct location, severely impaired LVEF, and the formation of a left ventricular aneurysm (LVA) [9]. However, these conventional markers are often identified concurrently with or after LVT has already formed, limiting their utility for early prevention.

There is a compelling clinical need for an early, accessible, and robust marker that can identify high-risk patients immediately after diagnosis [14]. The severity and complexity of coronary artery disease (CAD), as assessed by coronary angiography, may represent such an early marker. The Gensini score is a widely used angiographic tool that quantifies the extent and severity of coronary stenosis across the entire coronary tree [15]. A higher Gensini score reflects a greater plaque burden and is associated with poorer clinical outcomes post-MI [16,17]. While the SYNTAX score has been linked to LVT [18], the Gensini score assesses atherosclerotic burden more broadly. This study aimed to investigate the association between the Gensini score and LVT development in a cohort of patients with a first STEMI.

## 2. Materials and Methods

### 2.1 Study Design and Population

This single-center, retrospective observational study was conducted in accordance with the principles of the Declaration of Helsinki and the Strengthening the Reporting of Observational Studies in Epidemiology (STROBE) guidelines [19]. The study protocol was approved by the Ethics Committee of the First Hospital of Hebei Medical University (Approval No: 2018003101). Written informed consent was obtained from all participants.

**Fig. 1.**
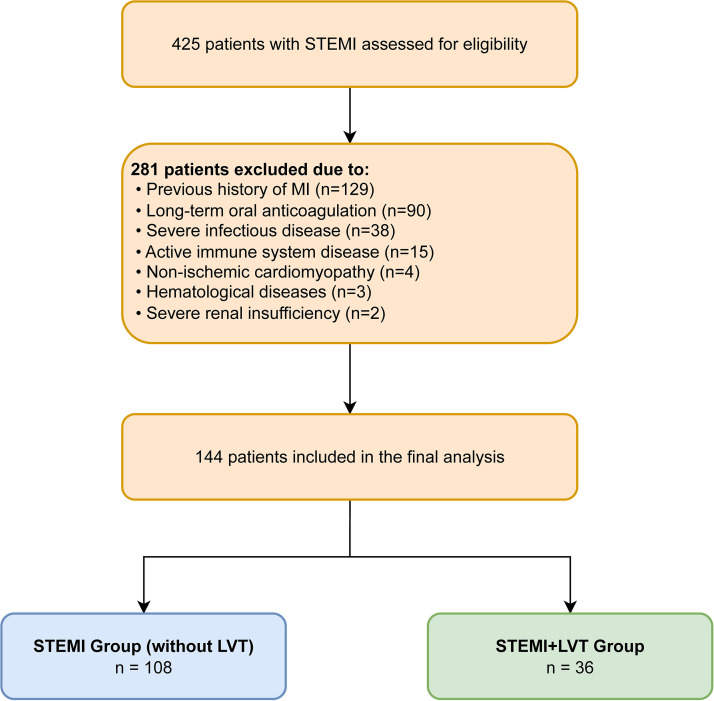
**Study flowchart**. Diagram illustrating the patient selection process according to the STROBE guidelines.

We retrospectively screened the medical records of 425 consecutive patients diagnosed with STEMI and admitted to the Cardiology Center of the First Hospital of Hebei Medical University between January 1, 2018, and January 1, 2021. Data for this analysis were extracted on August 15, 2021. The patient selection process is detailed in Fig. 1. Inclusion criteria were: (1) age ≥18 years; (2) diagnosis of a first acute STEMI based on the fourth universal definition of myocardial infarction, including characteristic symptoms, elevated cardiac troponin levels (at least one value above the 99th percentile upper reference limit), and new ST-segment elevation in at least two contiguous leads [20]; and (3) availability of complete medical, laboratory, echocardiographic, and angiographic records.

Exclusion criteria were: (1) previous history of myocardial infarction (n = 129); (2) ongoing long-term oral anticoagulation therapy for other indications (e.g., atrial fibrillation, mechanical valve replacement) (n = 90); (3) severe infectious diseases requiring systemic anti-infective treatment (n = 38); (4) active systemic immune diseases (n = 15); (5) known hematological disorders affecting coagulation or platelet function (n = 3); (6) non-ischemic cardiomyopathy (n = 4); and (7) severe renal insufficiency requiring hemodialysis (n = 2). These exclusions were applied to minimize confounding factors related to pre-existing thrombotic or bleeding risks. A final cohort of 144 patients was included: STEMI+LVT group (n = 36) and STEMI group (n = 108).

### 2.2 Data Collection and Definitions

Baseline data, including Killip classification [21], were collected. Venous blood samples were collected upon admission. Peak values of cardiac biomarkers (cardiac Troponin I (cTnI), Creatine Kinase-MB (CK-MB), B-type natriuretic peptide (BNP), C-reactive protein (CRP)) were documented. Echocardiographic examinations were performed using a Philips Ultrasound system (Philips Medical Systems, Andover, MA, USA) by experienced sonographers following ASE guidelines [22]. LVEF was calculated using the biplane Simpson’s method. LVT was defined as a discrete, echogenic mass adjacent to an akinetic or dyskinetic myocardial segment. TTE was performed during the index hospitalization or within one month of discharge. Coronary Angiography (CAG) was analyzed to calculate the Gensini score based on the degree of luminal stenosis and the functional significance of the coronary segment [15].

### 2.3 Statistical Analysis

Statistical analyses were performed using IBM SPSS Statistics for Windows, Version 26.0 (IBM Corp., Armonk, NY, USA). Continuous variables were compared using the independent samples *t*-test or Mann-Whitney U test. Categorical variables were compared using the Chi-square test. Variables were selected for the multivariable model based on a combination of statistical significance in univariate analysis (*p* < 0.05) and clinical relevance established in prior literature to avoid overfitting. A multivariable logistic regression model was constructed including the Gensini score, LVEF, LVA, and BNP, while explicitly adjusting for potential confounders: smoking history, alcohol consumption history, and Killip classification. Odds ratios (OR) for continuous variables were scaled for clinical interpretability: Gensini score (per 10-unit increase), BNP (per 100 pg/mL increase), and LVEF (per 5% increase). Receiver operating characteristic (ROC) curves were constructed to evaluate the discriminative ability of the Gensini score and the combined model, and the optimal cut-off value was determined using the Youden index. A two-sided *p*-value < 0.05 was considered statistically significant.

## 3. Results

### 3.1 Baseline Demographics and Clinical Characteristics

**Fig. 2.**
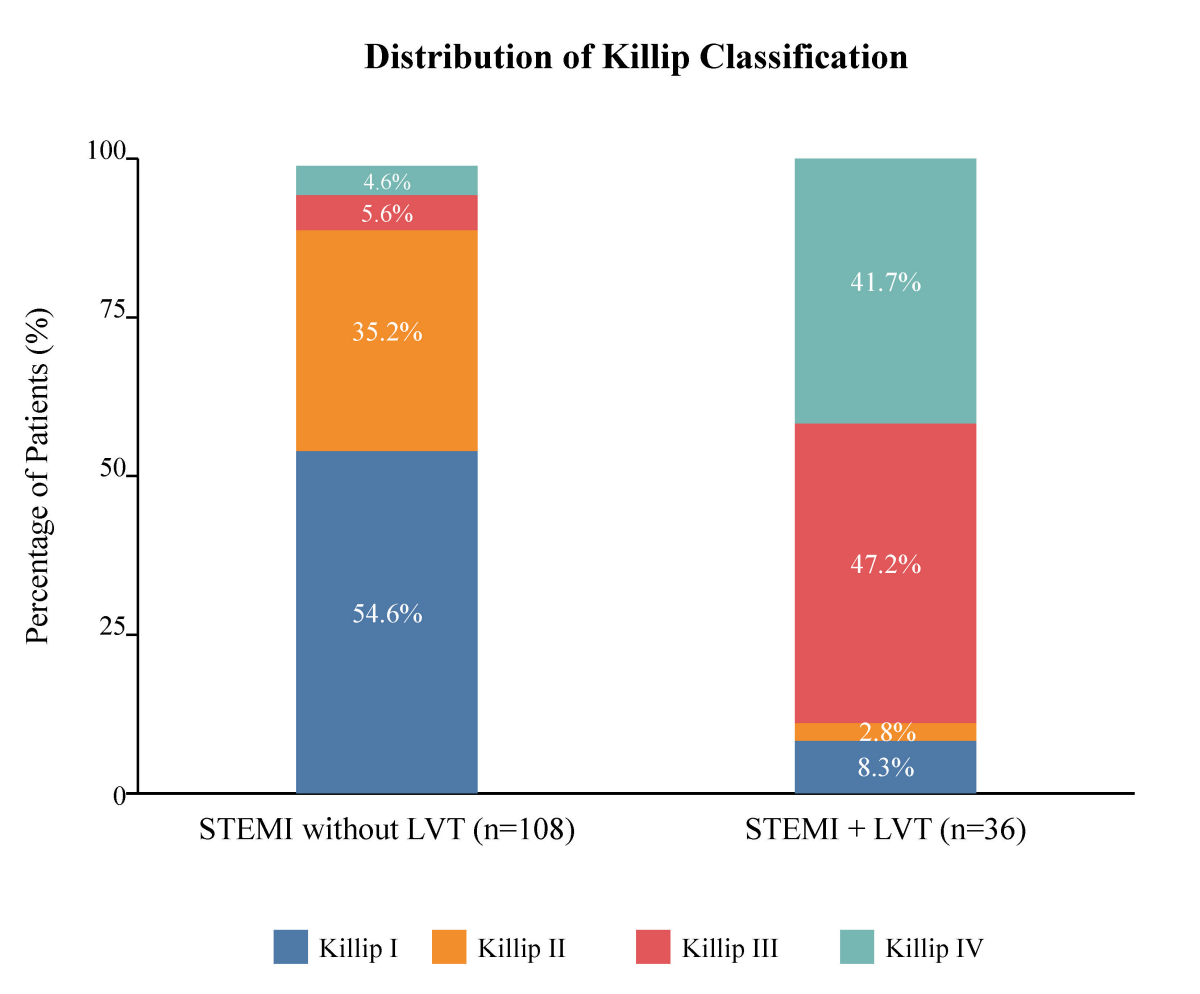
**Distribution of killip classification**. Stacked bar chart comparing the distribution of Killip classes I–IV between the STEMI without LVT and STEMI+LVT groups. Patients in the LVT group presented with significantly more severe heart failure (*p* < 0.001). STEMI, ST-segment elevation myocardial infarction; LVT, Left ventricular thrombus.

A total of 144 patients with a first STEMI were included, with 36 (25%) in the STEMI+LVT group and 108 (75%) in the STEMI group without LVT. The baseline characteristics of the study population are summarized in Table 1. The mean age and sex distribution were comparable between the two groups. However, patients in the STEMI+LVT group had a significantly higher prevalence of smoking (66.7% vs. 37.4%, *p* = 0.002) and alcohol consumption (75.0% vs. 24.1%, *p *< 0.001). Furthermore, patients who developed LVT presented with more severe heart failure, as indicated by a significantly higher distribution of Killip classes (*p *< 0.001) (Fig. 2). No significant differences were observed in BMI or history of hypertension, diabetes, or cerebrovascular disease.

**Table 1.** **Comparison of baseline demographics and clinical characteristics**.

Variable		STEMI Group (n = 108)	STEMI+LVT Group (n = 36)	Test statistic	*p*-value
Age (years), mean ± SD		56.45 ± 15.51	59.43 ± 10.73	t = –1.189	0.236
Male, n (%)		80 (74.1)	30 (83.3)	χ^2^ = 1.283	0.257
BMI (kg/m^2^), median [IQR]		25.86 [23.94, 28.08]	26.57 [23.64, 28.73]	Z = –1.534	0.125
Hypertension history, n (%)		58 (53.7)	15 (41.7)	χ^2^ = 1.565	0.211
Diabetes history, n (%)		29 (26.9)	11 (30.6)	χ^2^ = 0.158	0.691
Cerebrovascular disease history, n (%)		16 (14.8)	6 (16.7)	χ^2^ = 0.072	0.789
Smoking history, n (%)		40 (37.0)	24 (66.7)	χ^2^ = 9.600	**0.002**
Alcohol consumption history, n (%)		26 (24.1)	27 (75.0)	χ^2^ = 30.106	**<0.001**
Killip Classification				Z = –7.434	**<0.001**
	Killip I, n (%)	59 (54.6)	3 (8.3)		
	Killip II, n (%)	38 (35.2)	1 (2.8)		
	Killip III, n (%)	6 (5.6)	17 (47.2)		
	Killip IV, n (%)	5 (4.6)	15 (41.7)		

Data are presented as mean ± standard deviation, median [interquartile range], or n (%). BMI, body mass index; IQR, interquartile range; LVT, left ventricular thrombus; SD, standard deviation; STEMI, ST-segment elevation myocardial infarction. Bold values indicate *p* < 0.05.

### 3.2 Laboratory and Biomarker Findings

A comparison of key laboratory parameters is presented in Table 2. Patients in the STEMI+LVT group demonstrated evidence of more extensive myocardial injury, with significantly higher peak levels of cTnI (median 21.62 vs. 1.30 ng/mL, *p* < 0.001) and CK-MB (median 78.51 vs. 19.23 IU/L, *p* = 0.019). Markers of hemodynamic stress and inflammation were also significantly elevated in the LVT group, including BNP (median 1680 vs. 512 pg/mL, *p* < 0.001) and CRP (median 12.40 vs. 3.08 mg/L, *p* < 0.001). The LVT group also exhibited a more pronounced inflammatory response, with a higher neutrophil percentage (NE%) and a lower lymphocyte percentage (LY%). D-dimer (DD) levels, indicative of a prothrombotic state, were markedly higher in patients with LVT (median 1.530 vs. 0.120 mg/L, *p* < 0.001). Additionally, plasma Ca^2+^ concentration was significantly lower in the STEMI+LVT group (median 2.12 vs. 2.27 mmol/L, *p* = 0.001). No significant intergroup differences were found for WBC count, fibrinogen, mean platelet volume (MPV), or LDL-cholesterol.

**Table 2.** **Comparison of laboratory parameters**.

Variable	STEMI Group (n = 108)	STEMI+LVT Group (n = 36)	Z-value	*p*-value
Peak cTnI (ng/mL)	1.30 [0.09, 7.43]	21.62 [11.23, 73.21]	–4.208	**<0.001**
Peak CK-MB (IU/L)	19.23 [12.35, 46.76]	78.51 [18.76, 327.50]	–2.344	**0.019**
Peak BNP (pg/mL)	512 [37, 1240]	1680 [324, 2220]	–3.874	**<0.001**
Peak CRP (mg/L)	3.08 [1.33, 8.99]	12.40 [8.04, 32.06]	–3.624	**<0.001**
WBCc (10^9^/L)	8.20 [6.80, 11.10]	9.60 [7.80, 15.25]	–1.943	0.052
NE%	72.4 [65.6, 93.1]	84 [71.4, 87.3]	–2.134	**0.033**
LY%	20.7 [12.6, 27.4]	11.2 [8.7, 19.5]	–2.683	**0.007**
DD (mg/L)	0.120 [0.067, 1.015]	1.530 [0.139, 2.358]	–4.907	**<0.001**
FIB (g/L)	3.20 [2.72, 3.29]	3.39 [2.94, 4.03]	–0.739	0.460
MPV (fL)	8.70 [8.10, 9.63]	8.90 [8.55, 9.40]	–0.641	0.522
Plasma Ca^2+^ (mmol/L)	2.27 [2.20, 2.34]	2.12 [1.60, 2.23]	–3.221	**0.001**
LDL (mmol/L)	2.80 [2.28, 3.25]	3.05 [2.65, 3.37]	–1.536	0.125

Data are presented as median [interquartile range]. BNP, B-type natriuretic peptide; CK-MB, creatine kinase-MB; CRP, C-reactive protein; cTnI, cardiac Troponin I; DD, D-dimer; FIB, fibrinogen; LDL, low-density lipoprotein; LY%, lymphocyte percentage; MPV, mean platelet volume; NE%, neutrophil percentage; WBCc, white blood cell count. Bold values indicate *p* < 0.05.

### 3.3 Echocardiographic and Angiographic Findings

Echocardiographic parameters, detailed in Table 3, revealed profound differences in cardiac structure and function. Patients who developed LVT had significantly worse left ventricular systolic function, as evidenced by a lower median LVEF (41.0% vs. 59.0%, *p* < 0.001) and fractional shortening (FS) (19% vs. 31%, *p* < 0.001). The STEMI+LVT group also showed more advanced adverse remodeling, with larger left ventricular diameters (LVDd and LVDs) and a higher end-diastolic volume (EDV) (all *p* < 0.001). Critically, the incidence of LVA was dramatically higher in the LVT group (47.2% vs. 2.8%, *p* < 0.001). Mitral regurgitation was also more common and more severe in patients with LVT (*p* < 0.001).

**Table 3.** **Comparison of echocardiographic parameters**.

Variable		STEMI Group (n = 108)	STEMI+LVT Group (n = 36)	Test statistic	*p*-value
LVEF (%)		59.00 [48.25, 66.00]	41.00 [32.50, 44.00]	Z = –4.584	**<0.001**
Left atrial diameter (mm)		37.0 [33.3, 40.0]	39.0 [34.5, 41.5]	Z = –1.589	0.112
IVSD (mm)		11.5 [10.0, 12.0]	10.0 [9.0, 12.0]	Z = –2.063	**0.039**
LVDd (mm)		49.0 [46.0, 52.0]	58.0 [51.0, 63.0]	Z = –4.182	**<0.001**
LVDs (mm)		34.0 [30.0, 38.0]	47.0 [39.0, 55.0]	Z = –3.430	**<0.001**
EDV (mL)		113.0 [99.0, 128.8]	164.0 [126.0, 199.0]	Z = –3.370	**<0.001**
LVA, n (%)		3 (2.8)	17 (47.2)	χ^2^ = 44.594	**<0.001**
FS (%)		31.0 [25.0, 36.0]	19.0 [15.0, 23.0]	Z = –4.650	**<0.001**
Mitral Regurgitation				Z = –3.623	**<0.001**
	None, n (%)	27 (25.0)	1 (2.8)		
	Mild, n (%)	62 (57.4)	21 (58.3)		
	Moderate, n (%)	17 (15.7)	14 (38.9)		
	Severe, n (%)	2 (1.9)	0 (0)		

Data are presented as median [interquartile range] or n (%). EDV, end-diastolic volume; FS, fractional shortening; IVSD, interventricular septal thickness; LVA, left ventricular aneurysm; LVDd, left ventricular end-diastolic diameter; LVDs, left ventricular end-systolic diameter; LVEF, left ventricular ejection fraction. Bold values indicate *p* < 0.05.

**Fig. 3.**
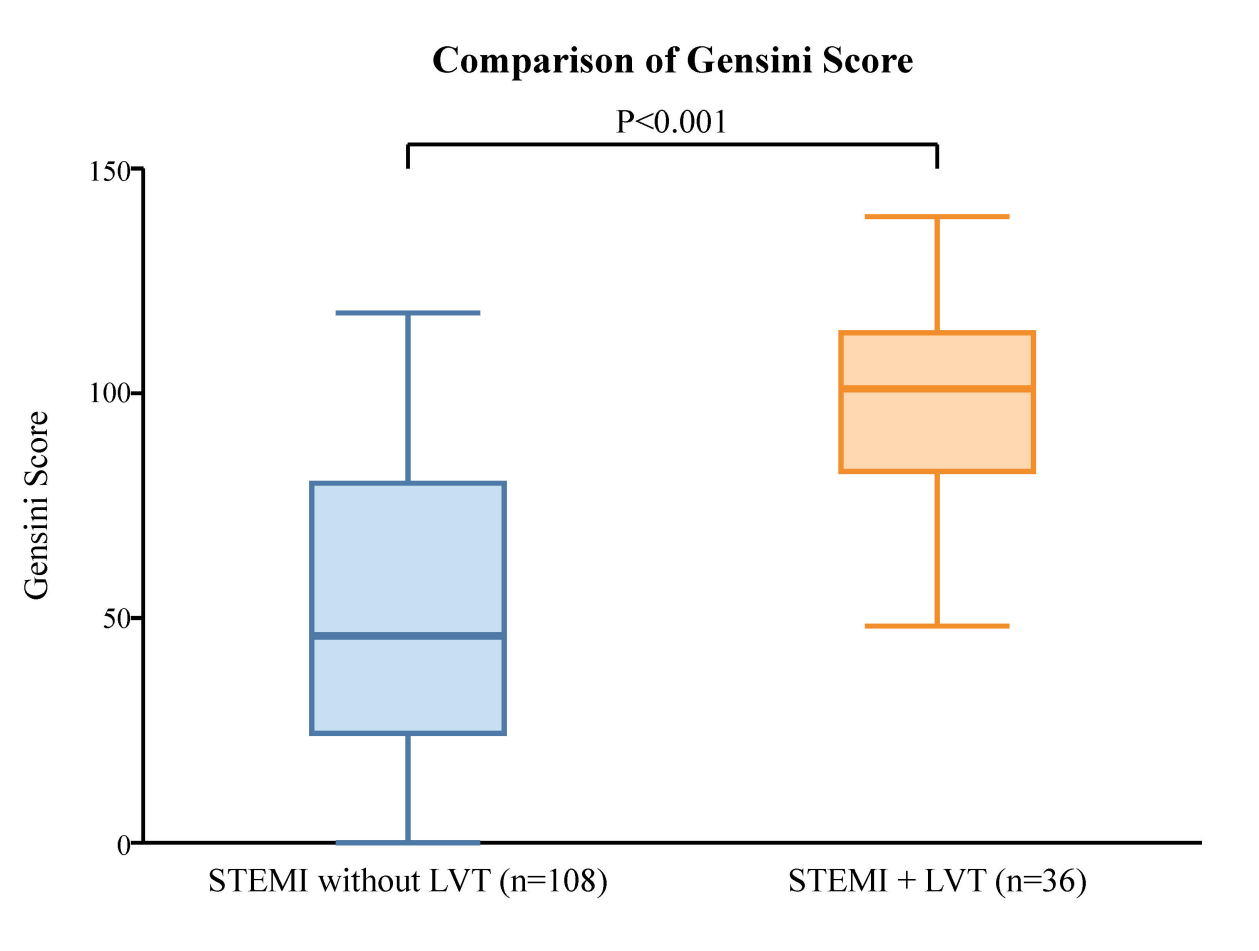
**Comparison of Gensini score**. Box plot showing the distribution of Gensini scores in the STEMI without LVT and STEMI+LVT groups. The median Gensini score was significantly higher in patients who developed LVT (*p* < 0.001). The box represents the interquartile range (IQR), the central line indicates the median, and the whiskers extend to 1.5 times the IQR.

The results of coronary angiography are presented in Table 4. While the culprit lesion was predominantly in the left anterior descending (LAD) artery in both groups, the proportion was slightly higher in the STEMI+LVT group (97.2% vs. 90.7%), though the difference was not statistically significant after adjustment. There were no significant differences in the involvement of the left main, left circumflex, or right coronary arteries, nor in the total number of diseased vessels. However, the overall burden of coronary atherosclerosis, as quantified by the Gensini score, was significantly higher in patients who developed LVT. The median Gensini score in the STEMI+LVT group was more than double that of the non-LVT group (101.0 [IQR 82.5–114.0] vs. 46.0 [IQR 24.3–80.0], *p* < 0.001) (Fig. 3).

**Table 4.** **Comparison of coronary angiography findings**.

Variable		STEMI Group (n = 108)	STEMI+LVT Group (n = 36)	Test statistic	*p*-value
LM lesion (≥50%), n (%)		14 (13.0)	3 (8.3)	χ^2^ = 0.589	0.443
LAD lesion (≥50%), n (%)		98 (90.7)	35 (97.2)	χ^2^ = 1.346	0.246
LCX lesion (≥50%), n (%)		55 (50.9)	17 (47.2)	χ^2^ = 0.148	0.701
RCA lesion (≥50%), n (%)		60 (55.6)	18 (50.0)	χ^2^ = 0.336	0.562
Number of Diseased Vessels				Z = –1.317	0.188
	1-vessel disease, n (%)	35 (32.4)	9 (25.0)		
	2-vessel disease, n (%)	39 (36.1)	11 (30.6)		
	3-vessel disease, n (%)	34 (31.5)	16 (44.4)		
	Gensini Score, median [IQR]	46.0 [24.3, 80.0]	101.0 [82.5, 114.0]	Z = –3.574	**<0.001**

Data are presented as median [interquartile range] or n (%). LAD, left anterior descending artery; LCX, left circumflex artery; LM, left main coronary artery; RCA, right coronary artery. Bold values indicate *p* < 0.05.

### 3.4 Association With Left Ventricular Thrombus Formation

In univariate analysis, higher Gensini score, lower LVEF, LVA, higher BNP, and higher CK-MB were associated with LVT. We focused our multivariable analysis on variables with strong biological plausibility: ischemic burden (Gensini score), hemodynamic stress (BNP), and mechanical stasis (LVA, LVEF). In the final multivariable logistic regression model (explicitly adjusting for smoking, alcohol, and Killip class), the following remained independently associated with LVT formation: elevated BNP (adjusted OR: 1.32 per 100 pg/mL, 95% CI: 1.19–2.05, *p* = 0.040), reduced LVEF (adjusted OR: 0.52 per 5% increase, 95% CI: 0.30–0.84, *p* = 0.010), presence of LVA (adjusted OR: 2.46, *p* = 0.006), and a higher Gensini score (adjusted OR: 1.47 per 10 units, 95% CI: 1.21–2.62, *p* = 0.015) (Table 5). In a post-hoc analysis controlling for anterior wall location (LAD culprit), the Gensini score remained significantly associated with LVT (OR 1.38 per 10 units, *p* = 0.021).

**Table 5.** **Univariate and multivariable logistic regression analysis for factors associated with LVT**.

Variable	Univariate analysis OR (95% CI)	*p*-value	Multivariable analysis adjusted OR (95% CI)	*p*-value
Gensini Score (per 10 units)	2.27 (2.10–3.11)	<0.001	1.47 (1.21–2.62)	0.015
LVEF (per 5% increase)	0.34 (0.21–0.54)	<0.001	0.52 (0.30–0.84)	0.010
LVA (Present)	31.32 (8.36–117.36)	<0.001	2.46 (1.28–4.67)	0.006
Peak BNP (per 100 pg/mL)	2.38 (1.48–3.81)	<0.001	1.32 (1.19–2.05)	0.040
Smoking History	3.40 (1.50–7.72)	0.003	1.82 (0.85–3.91)	0.124
Alcohol Consumption	9.46 (3.90–22.9)	<0.001	1.65 (0.72–3.78)	0.231
Killip Class (>II)	70.55 (20.99–237.08)	<0.001	1.55 (0.91–2.64)	0.095
Peak CK-MB	2.16 (1.54–3.02)	<0.001	---	---
Plasma Ca^2+^	0.89 (0.82–0.97)	0.043	---	---

Note: The final multivariable model included Gensini score, LVEF, LVA, and BNP, adjusting for Smoking, Alcohol, and Killip Class. Odds ratios for continuous variables are scaled as indicated.

### 3.5 ROC Curve Analysis

**Fig. 4.**
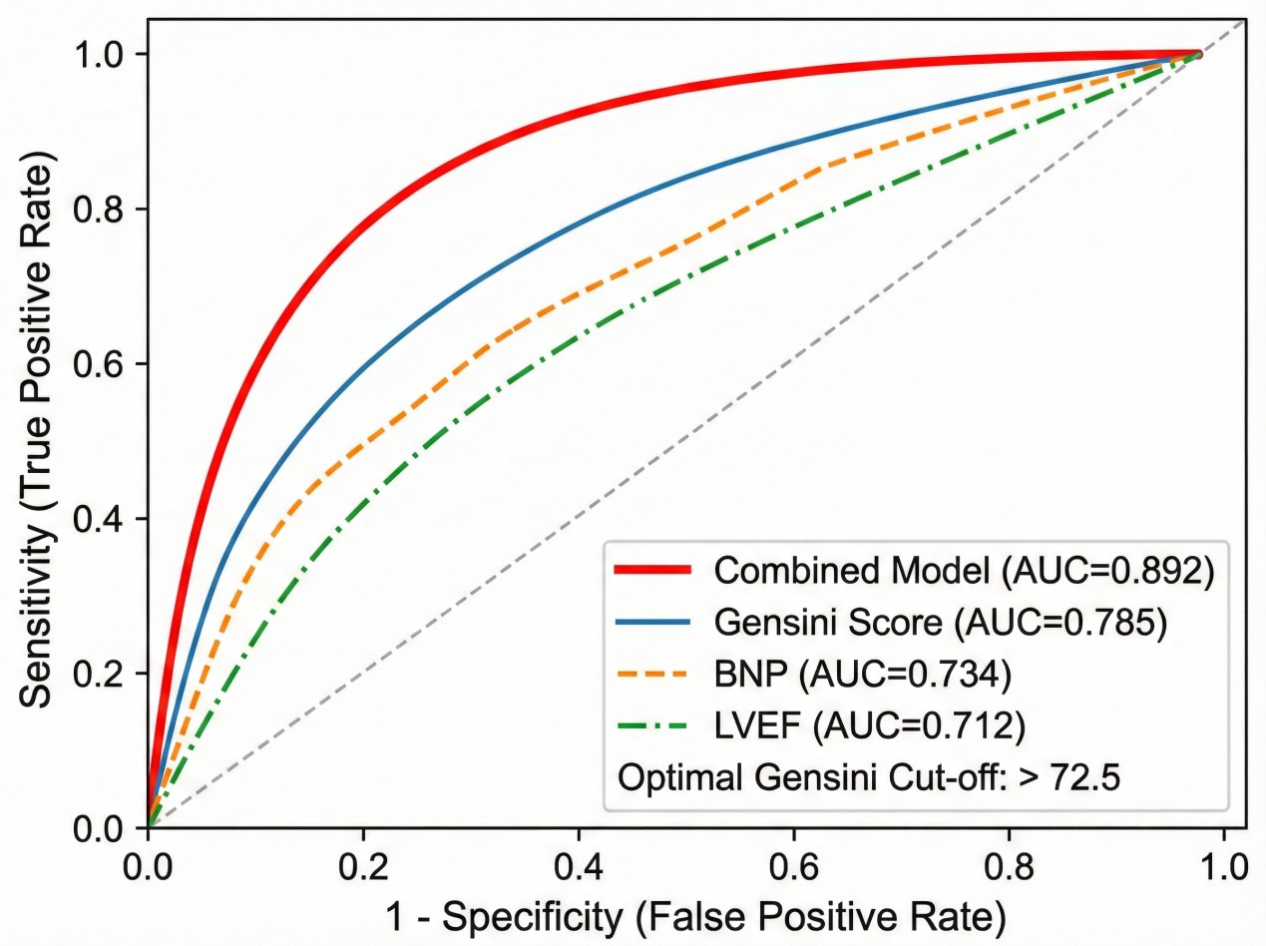
**Receiver operating characteristic (ROC) curves for the prediction of Left Ventricular Thrombus**. The curves represent the Gensini Score (AUC = 0.785), LVEF (AUC = 0.712), BNP (AUC = 0.734), and the Combined Model (AUC = 0.892).

Fig. 4 displays the ROC curves for the prediction of LVT. The Gensini score demonstrated good discriminative ability with an Area Under the Curve (AUC) of 0.785 (95% CI: 0.701–0.869). The Youden index identified an optimal cut-off value of 72.5 points, yielding a sensitivity of 77.8% and a specificity of 71.3%. The combined model (Gensini + LVEF + LVA + BNP) achieved the highest AUC of 0.892 (95% CI: 0.825–0.959).

## 4. Discussion

In this retrospective study of patients with a first STEMI, we identified that a higher Gensini score is independently associated with LVT formation, alongside elevated peak BNP, reduced LVEF, and the presence of LVA. Our principal finding is that the anatomical severity of coronary atherosclerosis, quantified by the Gensini score, serves as a valuable early risk marker.

The strong association between the Gensini score and LVT formation is pathophysiologically plausible. A higher Gensini score signifies a greater overall burden of coronary atherosclerosis, which is intrinsically linked to a larger infarct size, more profound endothelial dysfunction, and a more intense systemic inflammatory response—all key components of Virchow’s triad [10,23]. While anterior MI location is a well-known prerequisite for apical LVT [3], our data suggest that the extent of underlying multi-vessel disease further amplifies this risk, independent of the infarct location. Even after accounting for the functional consequences of the infarct (LVEF, LVA) and hemodynamic stress (BNP), the anatomical severity of CAD remained an independent risk marker. This aligns with previous work on the SYNTAX score [18], but the Gensini score is arguably more straightforward to calculate. Our ROC analysis suggests that a Gensini score >72.5 may warrant heightened vigilance.

Our study corroborates the pivotal roles of severe systolic dysfunction and adverse ventricular remodeling. The finding that a low LVEF and LVA are strong associated factors is consistent with literature [3,9,24]. Recent expert consensus documents emphasize the challenge of LVT prevention [3,4]. Current guidelines recommend screening for LVT with TTE in patients with anterior STEMI, but the timing is not well-defined. Our results suggest that a high Gensini score, available immediately after primary PCI, could identify a subset of patients at particularly high risk. Patients with high Gensini scores might benefit from more frequent screening or CMR, which has higher sensitivity [25].

## 5. Limitations

This study has limitations. First, the retrospective design and exclusion criteria introduce selection bias; our tertiary center population may have higher acuity, potentially explaining the relatively high LVT incidence (25%) compared to general registries. Second, echocardiographic parameters (LVEF, LVA) were measured during the index hospitalization, often concurrent with LVT diagnosis; thus, these represent an associated high-risk milieu rather than purely prospective predictors. Third, the number of events (n = 36) limits statistical power, although we used appropriate modeling techniques. Finally, causality cannot be inferred from this observational data.

## 6. Conclusion

The angiographic Gensini score is independently associated with LVT formation in patients with a first STEMI. A score >72.5, along with elevated BNP, low LVEF and LVA, characterizes a high-risk phenotype. Integrating the Gensini score into clinical assessment may help identify high-risk patients who could benefit from intensified imaging surveillance and targeted preventive strategies.

## Data Availability

The datasets generated or analysed during the current study are available from the corresponding author on reasonable request.

## Abbreviations

BMI, Body Mass Index; BNP, B-type Natriuretic Peptide; CAD, Coronary Artery Disease; CAG, Coronary Angiography; CI, Confidence Interval; CK-MB, Creatine Kinase-MB; CMR, Cardiac Magnetic Resonance; CRP, C-reactive Protein; cTnI, cardiac Troponin I; DD, D-dimer; EDV, End-Diastolic Volume; FS, Fractional Shortening; IQR, Interquartile Range; IVSD, Interventricular Septal Thickness; LAD, Left Atrial Diameter/Left Anterior Descending; LVA, Left Ventricular Aneurysm; LVDd, Left Ventricular End-Diastolic Diameter; LVDs, Left Ventricular End-Systolic Diameter; LVEF, Left Ventricular Ejection Fraction; LVT, Left Ventricular Thrombus; MI, Myocardial Infarction; MPV, Mean Platelet Volume; MR, Mitral Regurgitation; OR, Odds Ratio; PCI, Percutaneous Coronary Intervention; SD, Standard Deviation; STEMI, ST-segment Elevation Myocardial Infarction; TTE, Transthoracic Echocardiography.
